# Supplementary material for: Hepatic protein tyrosine phosphatase receptor gamma links obesity-induced inflammation to insulin resistance
Source: Nat Commun. 2017 Nov 28;8:1820. doi: 10.1038/s41467-017-02074-2 (PMC5703876; doi:10.1038/s41467-017-02074-2)
Supplement: Supplementary file 1 — Supplementary Information [file 41467_2017_2074_MOESM1_ESM.pdf]

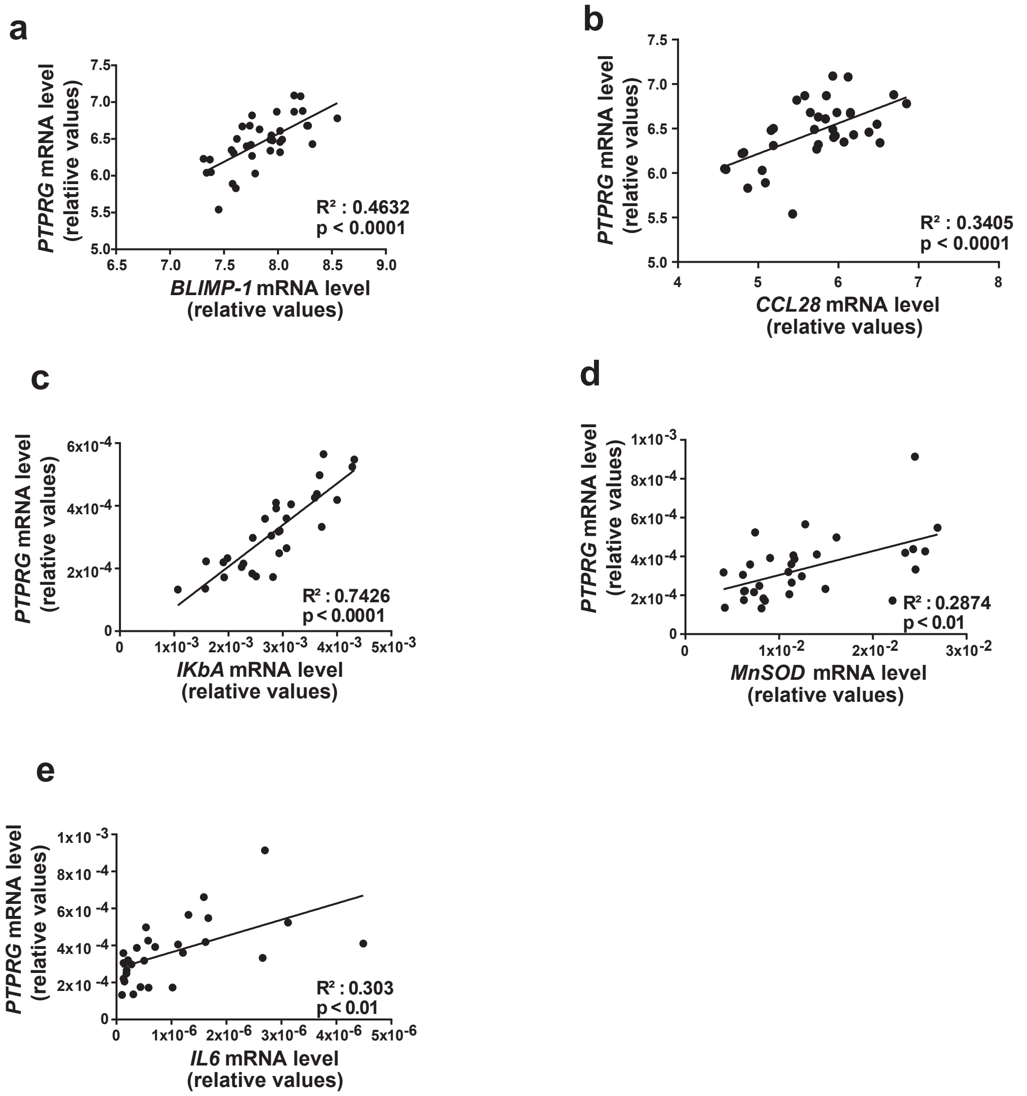

Supplementary Figure 1. **See also Figure 1.** Hepatic *PTPRG* expression correlates with inflammation and humans. Correlation between hepatic *PTPRG* mRNA expression and (**a**) *BLIMP-1* mRNA level, (**b**) *CCL28* mRNA level in patients with non-alcoholic steatohepatitis (NASH) (n=16) compared to healthy subjects (n=19) from public available microarray E-MEXP-3291. Correlation between hepatic *PTPRG* mRNA expression and (**c**) *IkBA* mRNA level, (**d**) *MnSOD* mRNA level, (**e**) *IL6* mRNA level in humans (n=33). Correlation analyses were performed by using the Spearman rank-correlation test.

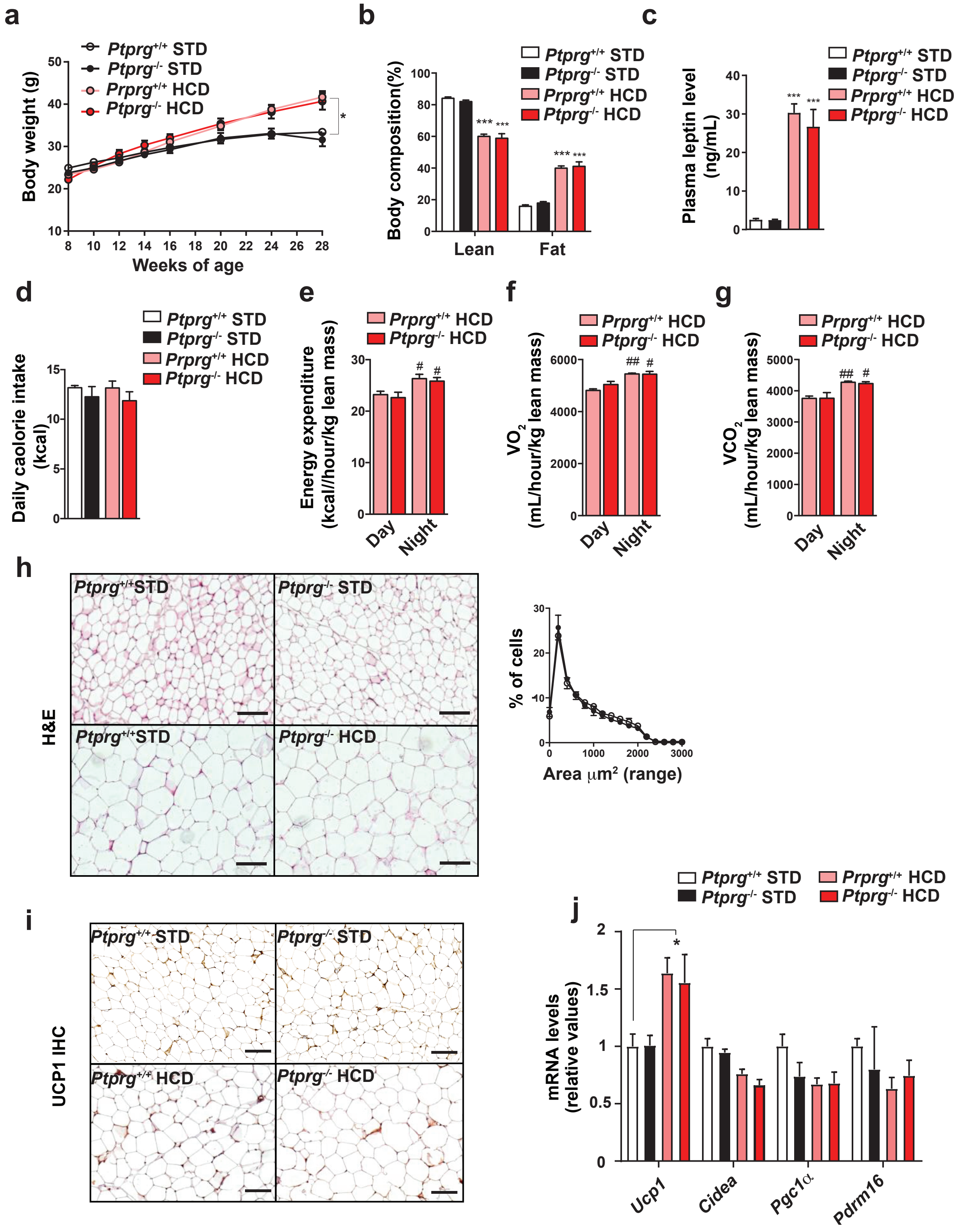

Supplementary Figure 2. See also Figure 2. PTPR-γ is not required for normal body weight homeostasis. (**a**) Body weight evolution over time in Ptprg$^{+/+}$ (n=8) and Ptprg$^{-/-}$ (n=8) mice fed either on a STD or a HCD. (**b**) Body composition of 28-week-old Ptprg$^{+/+}$ (n=8) and Ptprg$^{-/-}$ (n=8) mice fed either on a STD or a HCD. (**c**) Circulating leptin level in 28-week-old

Ptprg$^{+/+}$ (n=8) and Ptprg$^{-/-}$ (n=8) mice fed either on a STD or a HCD. (**d**) Daily calorie intake in 28-week-old Ptprg$^{+/+}$ (n=8) and Ptprg$^{-/-}$ (n=8) mice fed either on a STD or a HCD. (**e**) Energy expenditure, (**f**) O2 consumption, and (**g**) CO2 production in 26-week-old Ptprg$^{+/+}$ (n=8) and Ptprg$^{-/-}$ (n=8) mice fed on a HCD. (**h**) Hematoxylin and eosin (H&E) staining of perigonadal WAT of 28- week-old Ptprg+/+ (n=4) and Ptprg-/- (n=4) mice fed either on a STD or a HCD. The right panel represents adipocytes size distribution in perigonadal WAT of 28-week-old STD-fed Ptprg$^{+/+}$ (n=4) and Ptprg$^{-/-}$ (n=4) mice represented in open and black dots, respectively. (**i**) Representative photomicrographs of paraffin-embedded perigonadal WAT sections stained with hematoxylin and eosin (H&E) and treated for UCP1 immunohistochemistry (IHC). Tissues were collected from same STD- fed and HCD-fed mice as in h. Dark-brown staining represents UCP1-expressing brown adipocytes. (**j**) Relative mRNA level of "browning" markers in perigonadal WAT of 28-week-old Ptprg$^{+/+}$ (n=8) and Ptprg$^{-/-}$ (n=8) mice fed either on a STD or a HCD. Error bars represent SEM. Statistical analyses were done using two-tailed unpaired Student's t test. *P < 0.05, **P < 0.01, ***P<0.001: HCD vs. STD of same genotype; #P < 0.05, ##P < 0.01: night vs. day of same genotype. In (h) and (i): Scale bar = 100µm.

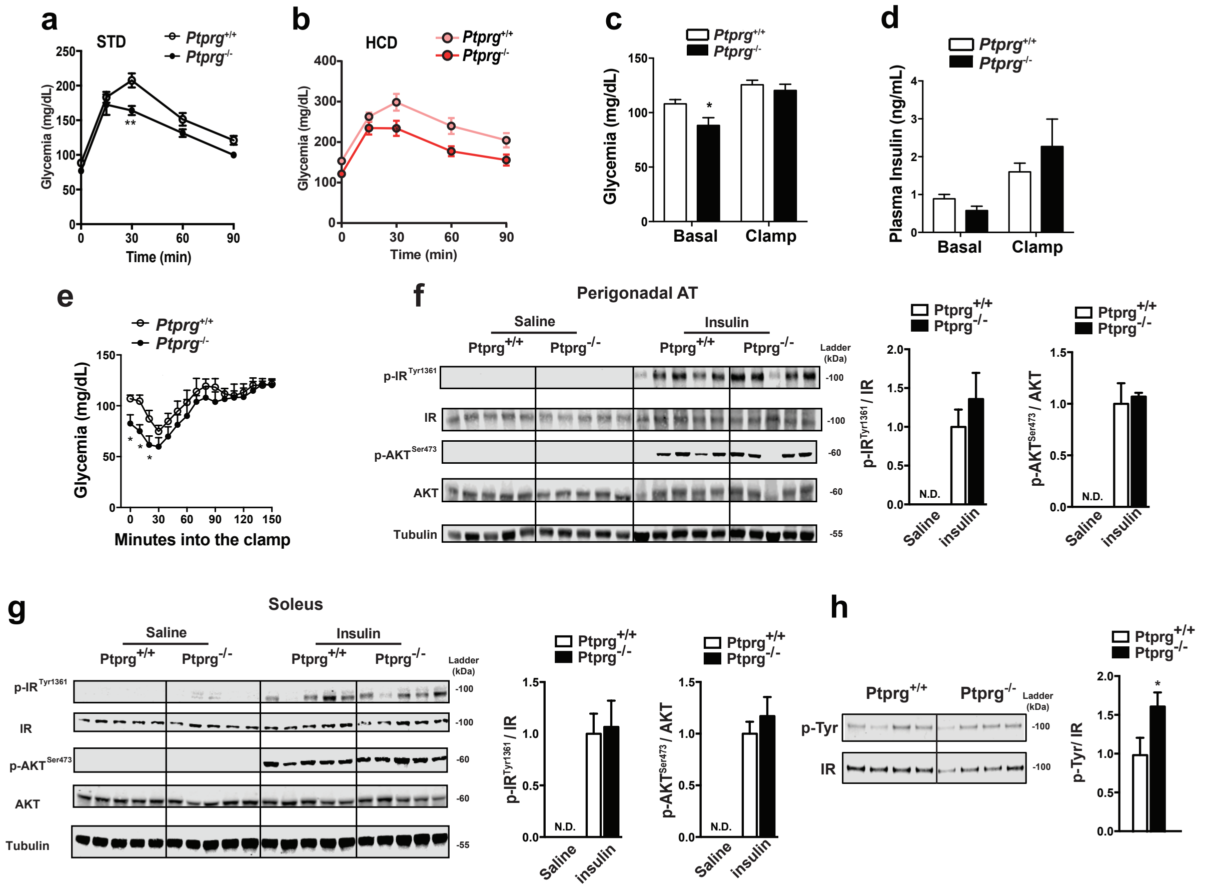

Supplementary Figure 3. See also Figure 2. Lack of PTPR-γ improves insulin sensitivity. (**a,b**) Glucose tolerance test and in 12-week-old STD-fed or 28-week-old HCD-fed Ptprg$^{+/+}$ (n=8) and Ptprg$^{-/-}$ (n=8) mice. (**c**) Circulating glucose and (**d**) insulin level in 12-week-old Ptprg$^{+/+}$ (n=6) and Ptprg$^{-/-}$ (n=6) mice fed on a STD in basal and clamp condition. (**e**) Circulating glucose level over time in same mice as in (**c-d**) during the clamp. Immunoblotting and relative quantification for the described total and phosphorylated proteins in (**f**) perigonadal adipose tissue and (**g**) soleus muscle of 10-week-old STD-fed Ptprg-/- and Ptprg+/+ mice 10 minutes after an intraperitoneal injection of either insulin (5 units/kg of body weight) or saline. (**h**) Immunoblotting and relative quantification of phospho-Tyrosine from liver protein lysate of 10-week-old STD-fed Ptprg$^{+/+}$ and Ptprg$^{-/-}$ after immunoprecipitation of the Insulin Receptor. Samples were obtained from mice 10 minutes after injection of insulin (5 units/kg of body weight) or saline. Error bars represent SEM. Statistical analyses were done using two-tailed unpaired Student's t test. *P<0.05; **P<0.01.

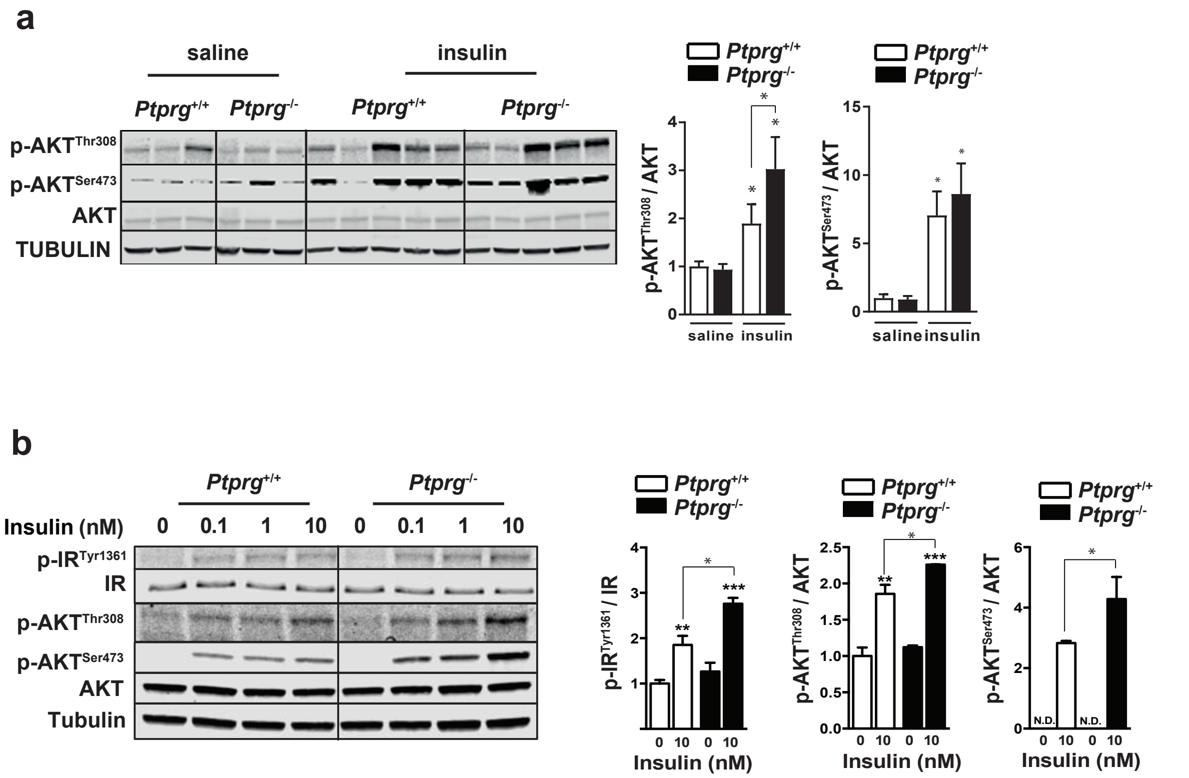

Supplementary Figure 4. See also Figure 2. Lack of PTPR-γ increases insulin signaling in insulin resistance context and in a cell autonomous manner. (**a**) Immunoblotting and relative quantification for the described total and phosphorylated proteins in the liver of 28-week-old HCD-fed Ptprg-/- and Ptprg+/+ mice 10 minutes after an intraperitoneal injection of either insulin (5 units/kg of body weight) or saline. (**b**) Representing immunoblotting and relative quantification for the described total and phosphorylated proteins in primary hepatocytes obtained from 10-week-old STD-fed Ptprg$^{+/+}$ and Ptprg$^{-/-}$ mice treated with the indicated concentration of insulin (n=3 for each group). Error bars represent SEM. Statistical analyses were done using two-tailed unpaired Student's t test. *P<0.05; **P<0.01; ***P<0.001

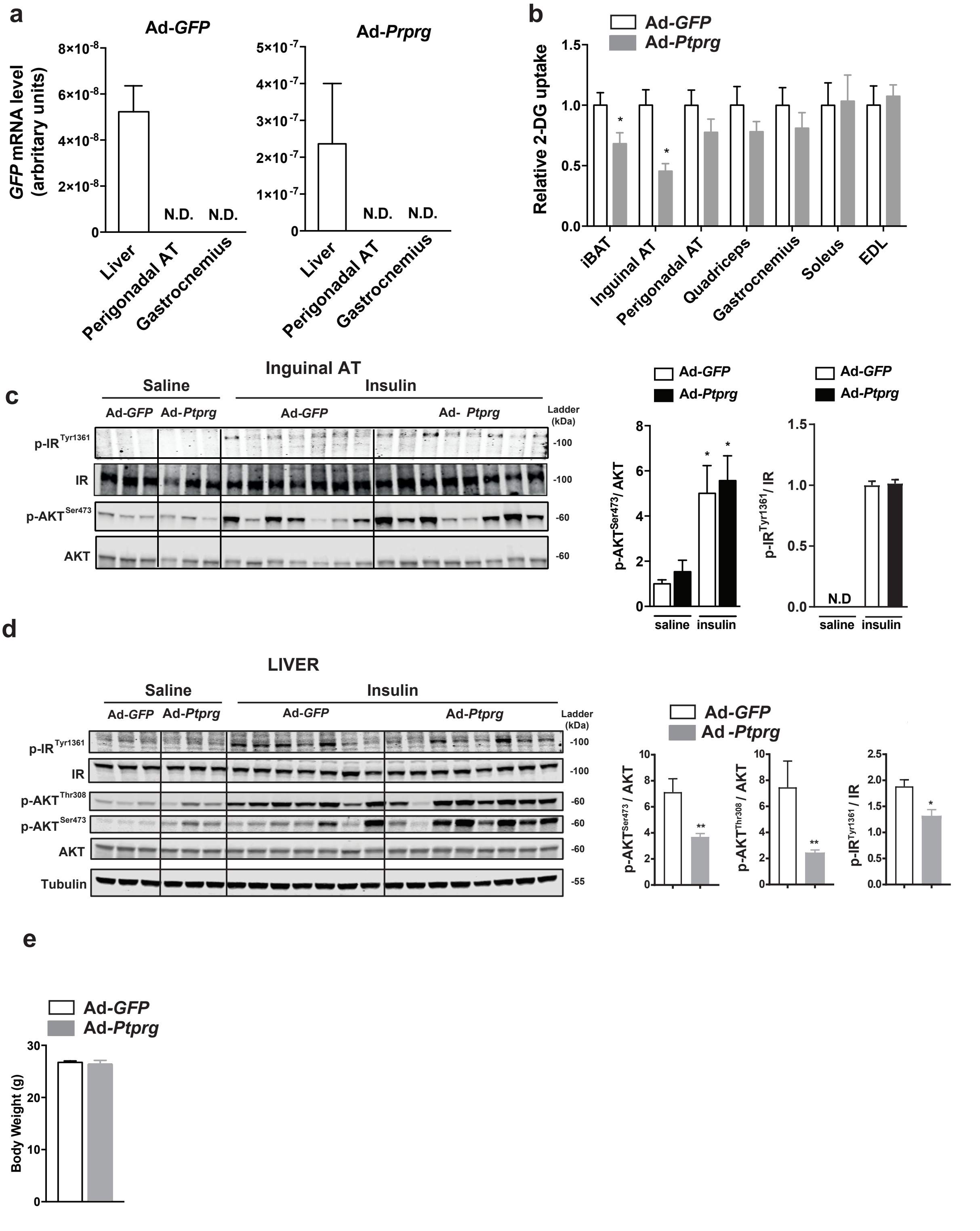

Supplementary Figure 5. See also Figure 3. Hepatic overexpression of PTPR-γ is sufficient to cause insulin resistance. Values indicated in **a-e** are obtained from 10-week-old FVB mice infected with either control adenovirus (Ad-*GFP*) (n=7) or adenovirus expressing mouse Ptprg (Ad-*Ptprg*) (n=8). (**a**) GFP mRNA expression in liver, perigonadal WAT, and

gastrocnenius of wild-type mice infected with adenovirus expressing *Ptprg* or *GFP*. (**b**) clamp peripheral tissues glucose disposal of wild-type FVB mice infected with adenovirus expressing *Ptprg* (n=7) or *GFP* (n=7) 14 days post infection. Immunoblotting and relative quantification for the described total and phosphorylated proteins in (**c**) inguinal adipose tissue and in (**d**) liver 10 minutes after an intraperitoneal injection of insulin (5 units/kg of body weight) or saline 14 days post infection. (**e**) Bodyweight of mice 14 days post infection. Error bars represent SEM. Statistical analyses were done using two-tailed unpaired Student's t test. *P<0.05; **P<0.01; ***P<0.001

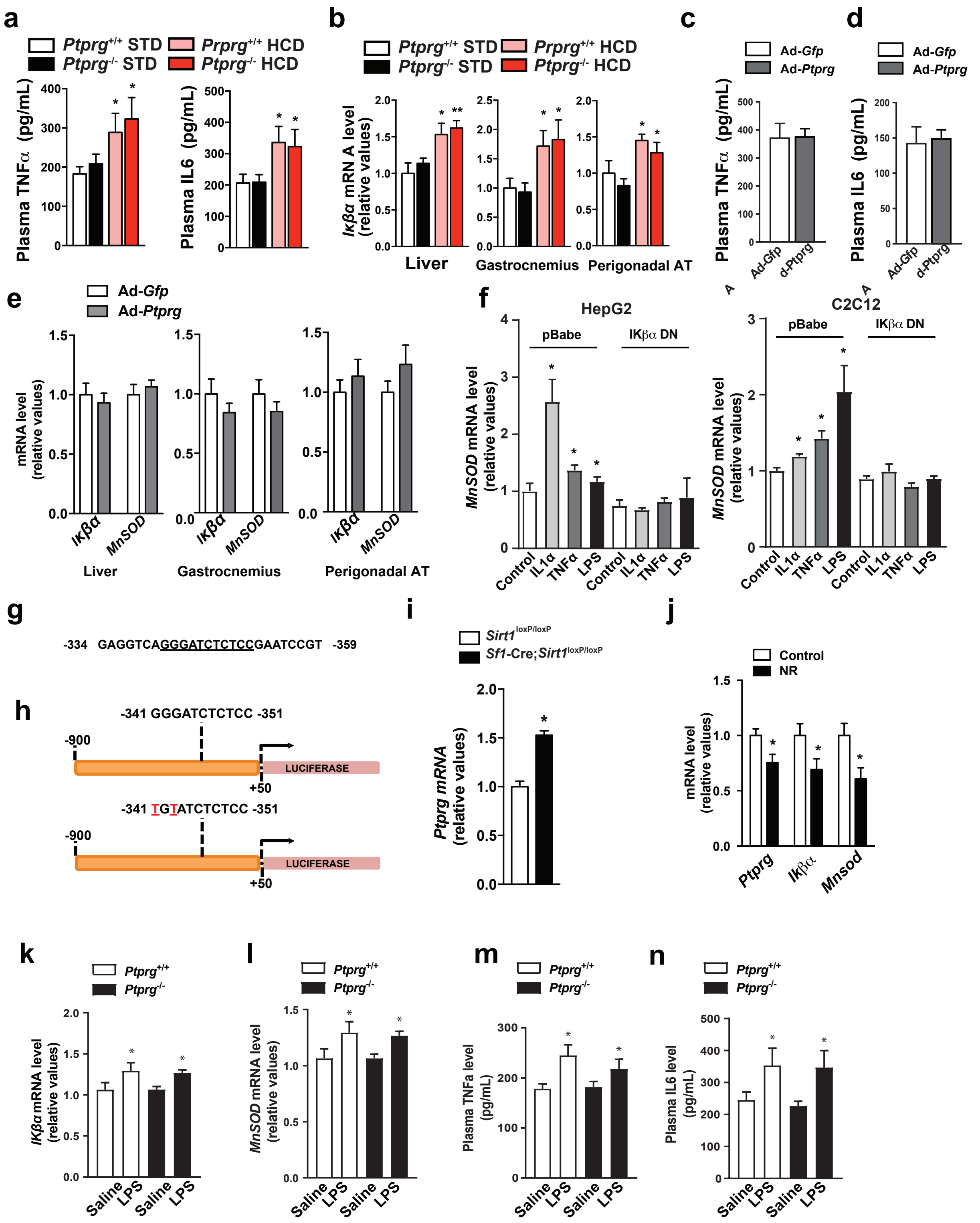

Supplementary Figure 6. See also Figure 4. NF-κB mediates inflammation-induced PTPR-γ expression. (**a**) Plasma levels of TNF-a and IL-6 in 30-week-old Ptprg$^{+/+}$ (n=8) and Ptprg$^{-/-}$ (n=8) mice fed either on a STD or a HCD. (**b**) *IκBα* mRNA expression in liver, gastrocnemius, and perigonadal WAT of 30-week-old Ptprg$^{+/+}$ (n=8) and Ptprg$^{-/-}$ (n=8) mice

fed either on a STD or a HCD. (**c**) Circulating TNF-α and (**d**) circulating IL6 of 10-week-old FVB mice infected with either control adenovirus (Ad-*GFP*) (n=7) or adenovirus expressing mouse Ptprg (Ad-*Ptprg*) (n=8) 10 days post infection. (**e**) *IκBα* and *MnSOD* expression in liver, gastrocnemius and perigonadal of 10-week-old FVB mice infected with either control adenovirus (Ad-*GFP*) (n=7) or adenovirus expressing mouse Ptprg (Ad-*Ptprg*) (n=8) 14 days post infection. (**f**) *MnSOD* mRNA level in HepG2 cells and C2C12 cells treated as indicated. (IκBα DN expresses a mutant "super-repressor" allele of *IκBα* harboring two amino acid substitutions: Serine 32 and 36 are substituted in Alanine). (**g**) Putative consensus motif for binding of NF-κB on Ptprg promoter region. (**h**) Scheme of the *Ptprg* promoter region (spanning 900bp before and 50bp after the 5'UTR starting site) cloned (between KpnI and HindIII restriction sites) in pGL2 basic vector (Promega) upstream the Firefly luciferase expression cassette and added mutations (G to T) in the NF-κB putative consensus motif. (**i**) *Ptprg* mRNA level obtained by QPCR from dissected VMH from 10-week old Sirt1loxP/loxP and Sf1-Cre;Sirt1loxP/loxP mice (n=3/group) fed on a STD diet. (**j**) *Ptprg*, *IκBα*, and *MnSOD* mRNA expression level in liver of HCD mice treated with the NAD+ precursor and SIRT1 activator nicotinamide riboside. (**k-l**) Hepatic mRNA levels of inflammation markers and (**m-n**) plasma level of inflammation cytokines of in 12-week old mice Ptprg$^{+/+}$ (n=6) and Ptprg$^{-/-}$ (n=6) treated 28 days with LPS.. Error bars represent SEM. Statistical analyses were done using two-tailed unpaired Student's t test. *P<0.05; **P<0.01; ***P<0.001.

Supplementary Table 1. Values representing relative detectable level of phosphorylation of tyrosine on specific residues obtained with Pamgene array technology (see Methods). Results are obtained from liver lysate of 4 $Ptprg^{+/+}$ mice and 4 $Ptprg^{-/-}$ mice and are represented as $\log_2$ of normalized values.

Tyrosine residue	Ptprg+/+ 1	Ptprg+/+ 2	Ptprg+/+ 3	Ptprg+/+ 4	Ptprg-/- 1	Ptprg-/- 2	Ptprg-/- 3	Ptprg-/- 4
41_654_666	7.7933979	7.74612761	7.81567955	7.58151341	8.12428761	8.20306683	8.12321377	7.77909374
ANXA1_14_26	6.22507572	5.68290186	5.46110106	5.80526304	6.72655869	6.8043623	6.41377163	6.42522001
ART_004_EAIYAAPFAKKKXC	9.2889185	8.99711037	9.30204392	9.34306812	9.82528019	9.75090313	9.73054028	9.65759468
CD79A_181_193	11.4459591	11.6075573	11.4261732	11.4331465	12.207571	12.1772614	11.8675966	11.6400147
CDK2_8_20	8.30254841	8.22515774	8.19983006	8.04652691	8.65589809	8.78430748	8.59003639	8.76052856
CTNB1_79_91	6.5489893	5.63933039	5.56939697	6.00029755	6.790905	6.45282793	6.64459086	6.1076169
EFS_246_258	9.74457073	9.86884308	9.69571114	9.76720715	10.3884077	10.6432819	10.3061247	9.95673561
EGFR_1103_1115	6.07528257	5.01918173	5.80380201	5.93639374	6.2268939	6.19194841	6.26809406	5.87463903
EGFR_1165_1177	6.58969021	6.37723494	6.74799013	6.25923681	6.64622259	6.81358767	6.91135263	6.12449217
ENOG_37_49	9.44668198	9.52750301	9.30922794	9.279006	10.0497141	10.1719198	9.78141213	9.60414028
EPHA1_774_786	7.30079699	7.22185135	6.72037649	7.03232145	7.24805307	7.7129097	7.32917452	7.4538064
EPHA2_765_777	7.28761673	7.25466347	6.82443667	7.04391956	7.37842989	7.84675598	7.45659828	7.55440044
EPHA4_589_601	6.22616673	5.62285328	6.11391926	0	6.45220614	6.2663269	6.38962507	5.94481421
EPHA7_607_619	6.91767025	6.6554327	6.68324614	6.80825377	7.48574591	7.35600758	7.22876406	6.82899284
EPHB1_771_783	6.55305338	6.31020975	6.24638653	6.1040597	7.28935766	7.38055515	6.97310448	6.90000105
EPOR_361_373	6.99076223	6.19826412	6.63037348	6.66565752	7.26920605	7.28622198	7.08600092	6.72372866
EPOR_419_431	6.63558483	6.48448467	6.33974934	6.57091522	6.95610476	6.98819161	6.92664862	6.73373795
FAK1_569_581	7.08539963	6.63558483	6.85344505	6.90772963	6.80484963	7.13194418	7.03688908	7.04659891
FAK2_572_584	7.92006636	7.80926132	7.9432559	7.87265444	8.41064739	8.43468285	8.22824669	7.58279181
FER_707_719	6.88395739	6.60263252	6.68694973	6.79850531	7.48224878	7.41350031	6.90273333	6.95369148
FES_706_718	7.35051394	7.35134745	7.35317993	7.29976082	8.29727936	8.19491482	7.91443586	7.66462517
FGFR2_762_774	6.62293577	6.24531078	6.27197409	6.56747532	6.98754835	7.05009508	6.93267727	6.85509157
FGFR3_753_765	6.21741343	5.25271893	5.1460619	5.72210503	6.4808321	6.6149044	6.05945158	6.36737776
FRK_380_392	8.77613545	8.69109821	8.5051527	8.62292862	9.35459042	9.51136875	9.12878227	8.86229801
JAK1_1015_1027	6.62321186	5.63987637	6.25603485	5.90175486	7.07109785	7.14200115	6.91270876	6.4214139
JAK2_563_577	6.21375084	5.17949629	6.20934296	5.88619137	6.88303518	6.73705912	6.63064861	6.24351549
K2C6B_53_65	7.58591223	7.53647804	7.61577892	7.68764973	7.66261149	7.91826677	7.82110596	7.6363101
K2C8_425_437	6.25282621	5.96325588	6.07285833	6.36176157	6.44878244	6.37526894	6.19678068	6.48630762
KSYK_518_530	6.5094986	5.63276196	6.3026433	6.01004505	6.55508089	6.63503695	6.62734795	6.39513397
LAT_249_261	6.22216177	5.51447964	5.7717762	6.23883724	6.90682268	7.07997799	6.73501635	7.0069375
LCK_387_399	6.65759134	6.45127344	6.54257917	6.42363548	7.3813715	7.11142111	7.1249404	6.9120307
MBP_198_210	7.02857351	6.24207735	6.75178385	6.72630167	6.95193338	6.83948898	6.75883913	6.73450518
MET_1227_1239	7.3616457	6.90296078	7.33002043	7.15752792	7.32238722	7.37302113	7.47078562	6.8772583
MK07_211_223	6.24171782	5.68184185	6.22507572	6.09213877	6.11705971	6.50530958	6.17885733	5.88111973
MK10_216_228	7.5910039	7.24374867	7.40244103	7.49407435	7.39388847	7.55019474	7.55425596	7.65924883
MK12_178_190	6.23775578	5.81544972	6.32726049	6.21154881	6.62734795	6.22979832	6.49448204	5.84653139
NTRK2_696_708	7.52366543	7.18827629	7.04309416	7.16076565	7.30734253	7.51595259	7.42098522	7.29664803
P85A_600_612	7.21929646	7.19776392	6.94929266	7.19423676	7.58577061	8.14587879	7.50415564	7.64299822
PAXI_111_123	8.12096691	8.003685	8.023592	7.9623723	8.82028103	8.81188679	8.40752411	8.31960392
PAXI_24_36	7.70366859	7.6922636	7.58364344	7.65398741	8.41902161	8.35885429	8.14000511	7.7746253
PDPK1_2_14	7.56880951	7.16304684	7.04536295	7.25127268	7.96955776	7.80537272	7.67105055	7.59057999
PDPK1_369_381	7.0574708	6.70036125	6.82563686	6.80046034	7.18041611	7.46031284	7.33323145	7.10530567
PECA1_706_718	7.72916555	7.33407497	7.45969439	7.54437399	7.9705348	8.16620541	7.88156986	7.99698782
PGFRB_1002_1014	5.54919243	5.03421259	5.1157651	5.04661942	6.44722366	6.2702117	6.16298962	5.52397633
PGFRB_572_584	7.97821951	7.6851387	7.72826719	7.8487649	8.50859451	8.68535709	8.36802864	7.86333323
PGFRB_709_721	6.33739471	5.95230722	6.29330254	6.23667336	6.67287874	6.45624209	6.39836454	6.35778427
PGFRB_768_780	6.23775578	5.93372393	6.06921434	5.74258614	6.55594921	6.34846115	6.25852585	5.8685956
PLCG1_764_776	8.86772728	8.7238512	8.57541084	8.72603798	9.28368664	9.4594202	9.09667587	8.92887878
PP2AB_297_309	6.87795258	6.30160856	6.77732039	6.41536713	6.63722658	6.78869057	6.74011803	6.73680401
PRRX2_202_214	7.27589607	6.86306429	6.92194176	7.11063337	7.23726845	7.43772459	7.25697851	7.17213488
RAF1_332_344	8.1133194	7.98629284	7.84711075	8.0365057	8.0334959	8.35902023	8.16421413	8.01793289
RASA1_453_465	8.03691959	8.0690012	7.85618496	8.00049973	8.43986607	8.45499325	8.35353851	8.1510601
RET_1022_1034	7.55483484	7.39194489	7.2079258	7.17043543	7.93159628	8.11440086	7.65924883	7.57453299
RON_1346_1358	6.42109633	5.07273674	5.32104302	5.53105784	6.62210703	6.3464551	6.27584362	5.78121138
RON_1353_1365	6.57976437	5.8852706	6.40416145	6.31295157	6.63257027	6.72115088	6.78942919	6.18523073
SRC8_CHICK_476_488	9.06592178	9.00529099	8.74170208	8.89714336	9.44707108	9.73194981	9.42550087	9.33668327
SRC8_CHICK_492_504	8.89760017	8.95132351	8.64049721	8.87394905	8.9178896	9.66566086	9.16175938	9.26314545
TEC_512_524	6.95610476	6.42014313	7.0395813	6.69090748	7.04762793	7.16019487	6.97006798	6.38865089
TYRO3_679_691	8.09551621	7.67438555	7.84888268	7.87833452	7.81567955	7.853127	8.03172875	7.93271065
VGFR1_1326_1338	6.0053916	5.82939959	5.92971087	5.27468348	6.58175468	6.46735954	6.09731674	5.99005604
VGFR2_989_1001	7.46201229	7.06562185	7.03751087	7.19980192	7.75699043	8.07345486	7.84959078	7.66877794
ZAP70_485_497	7.185287	6.98969173	6.86680079	6.98840618	7.4678669	7.61021709	7.31915236	6.90091229
ZBT16_621_633	7.0052433	6.30471087	6.65462256	6.89200163	6.89039612	6.87029505	6.9102211	6.88878918

Supplementary Table 2. Sequence of primers used for real-time–Qpcr and ChIP assay. List of antibodies used for Western Blotting and Immunohistochemistry procedures.

Gene	Forward	Reverse
18s	ACCGCAGCTAGGAATAATGGA	GCCTCAGTTCCGAAAACCA
Ptprb	GTGGTACCCAGAAGTGCCC	TCTGCCACTCCAGTCTGCAC
Ptprc	CTTCAGTGGTCCCATTGTGGTG	TCAGACACCTCTGTCGCCTTAG
Ptprg	GGCTTTCCCCGACGTTCCCG	TCGTTGGGGACCACCCGAAGG
Ptprj	CAAATGCGTGGAGCAGGGAA	CATCGCCACAGTTATGTCTCCA
Ptprk	ATGCAGAAGACCCCCTTTCC	ACTGTGGTTCTCATCTAACACGG
Ptpn12	GGAGGTTCATCCAGAGGGTC	TCTTCTCAATCGCATGAAGTCC
Ptpro	GCACCTGCCTAGGGGAAC	CGTCGTAACAATCTTGAGCAGC
Gfp	GAAGCAGCACGACTTCTTCAA	AAGTCGATGCCCTTCAGCTC
Luciferase	CCTCTAGAGGATGCAACCGC	GTGATGTTCACCTCGATATG
Mnsod	GTAGGGCCTGTCCGATGATG	CGCTACTGAGAAAGGTGCCA
Mnsod (human)	CGACCTGCCCTACGACTACCG	TGACCACCACCATTGAACTT
Ikba	CGGAGGACGGAGACTCGTT	CCATGGTCAGCGGCTTCT
Ikba (human	AATGCTCAGGAGCCCTGTAA	CTGTTGACATCAGCCCCACA
Ucp1	GAGGTGTGGCAGTGTTCATTG	GGCTTGCATTCTGACCTTCA
Cidea	GGTTCAAGGCCGTGTTAAGG	CGTCATCTGTGCAGCATAGG
Pgc1a	GAATCAAGCCACTACAGACACCG	CATCCCTCTTGAGCCTTTCGTG
Pdrm16	CAGCACGGTGAAGCCATTC	GCGTGCATCCGCTTGTG
**CHiP**	**Forward**	**Reverse**
PTPRG prom NFKB binding site	CCTTACGGAAGTCCCCTCTCC	AAAGGGTGGTCTGGTTGGAAT
	**Antibody**	
**Name**	**Supplier**	**Reference**
Akt	Cell Signaling	#2920
Phospho Akt Ser 473	Cell Signaling	#4060
Phospho Akt Thr 308	Cell Signaling	#9275
IR	Santa Cruz Biotechnology	Sc-711
Phospho IR 1361	Abcam	Ab60946
Tubulin	Abcam	Ab6160
UCP1	Pierce	PA1-24894
